# A new gene on *C. elegans* chromosome V

**DOI:** 10.17912/micropub.biology.000496

**Published:** 2021-11-03

**Authors:** Anusha Iyengar, Stavros Diamantakis, Adam Norris

**Affiliations:** 1 Southern Methodist University, Dallas, TX, USA; 2 European Molecular Biology Laboratory, European Bioinformatics Institute, Wellcome Trust Genome Campus, Hinxton, Cambridge CB10 1SD, UK

## Abstract

*C. elegans *was the first animal to have its genome completely sequenced. In the decades since, the genome continues to be actively curated, annotated, and improved. Here we report the discovery of a new gene in a region of the genome that is currently not associated with any annotated gene or feature. We present RNA-seq and RT-PCR evidence that this gene is expressed at detectable levels, and that it is alternatively spliced. The new gene (*Y97E10C.2*) shares an operon with two upstream genes. We provide RNA-seq and RT-PCR evidence for a missing exon in the upstream gene* T05B11.7*, as well as an alternatively-spliced exon.

**Figure 1. A new gene, a longer gene, and a larger operon on chromosome V:**
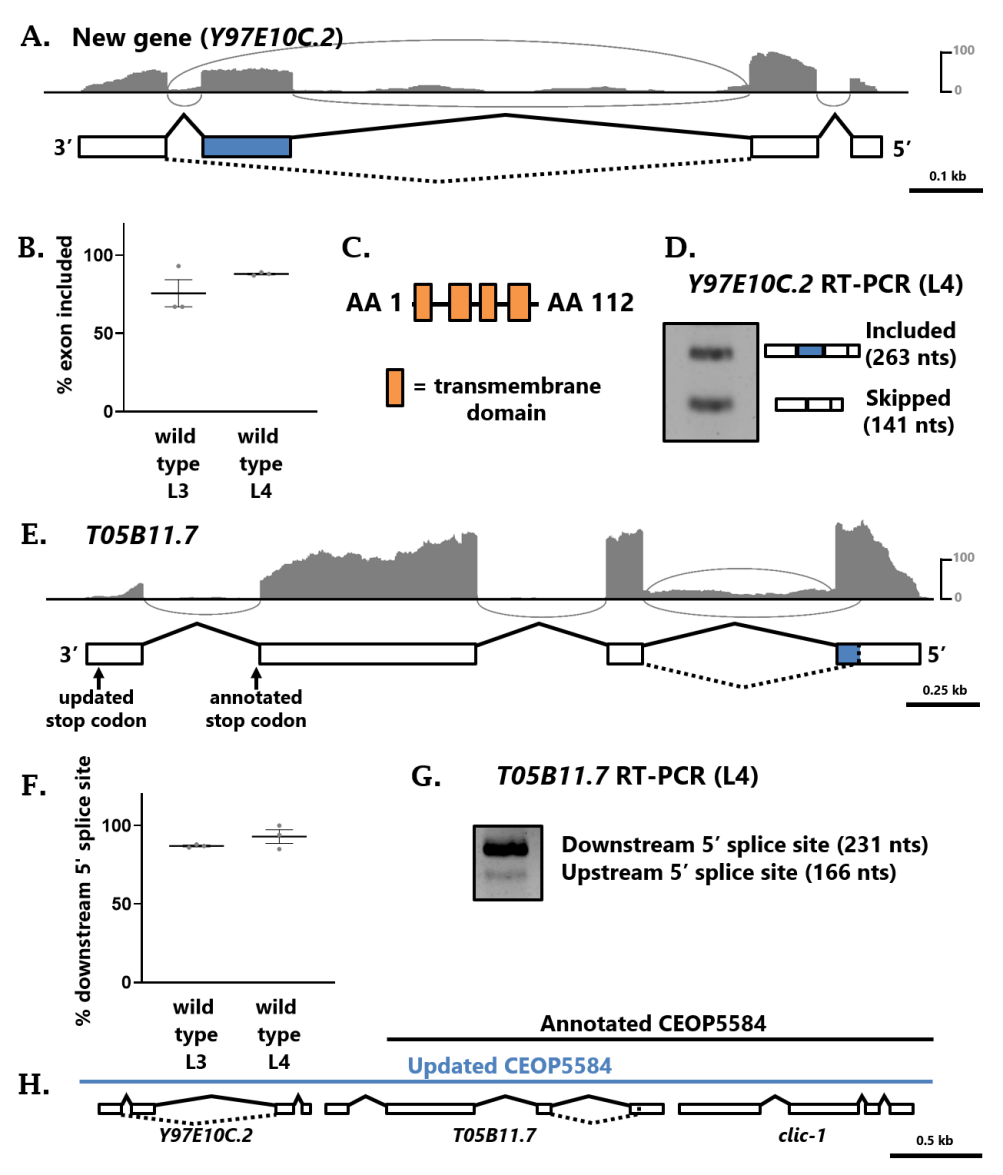
(A) Gene model for new gene *Y97E10C.2* with RNA-seq evidence from wild-type L4-staged whole worms. Vertical scale bar = number of reads. Alternative exon highlighted in blue. (B) % exon included of the alternative third exon in *Y97E10C.2*, according to RNA-seq performed in biological triplicates at the L3 or L4 stage of wild-type whole worms. (C) Predicted protein domain structure of the major *Y97E10C.2a* isoform. (D) RT-PCR confirms alternative splicing of exon three in *Y97E10C.2.* (E) Gene model for *T05B11.7*, as in panel A. The alternative 5’ splice site in exon one, yielding transcripts of either 732 or 924 nts, is highlighted in blue. (F) % usage of the downstream 5’ splice site of *T05B11.7* exon one, according to RNA-seq performed in biological triplicates at the L3 or L4 stage of wild-type whole worms. (G) RT-PCR confirms the usage of the alternative 5’ splice site, albeit at low levels*.* (H) CEOP5584 now includes both the fourth exon of *T05B11.7* and the new gene *Y97E10C.2.*

## Description

In the decades since the *C. elegans* genome sequence was completed (C. elegans Sequencing Consortium, 1998), ongoing curation efforts continue to further improve and annotate the genome (Harris *et al.*, 2020). In the course of searching for alternatively-spliced genes using RNA-seq data, we happened upon an alternative splicing event in a region of the *C. elegans* genome with no associated gene or feature annotation. Upon closer inspection, we identified RNA-seq support for an unannotated four-exon gene, with the third exon being alternatively spliced (Fig 1A). The putative coding sequences of this gene encode proteins of either 86 or 122 amino acids, depending on the splicing choice. The longer isoform (exon three included) is the major isoform according to our RNA-seq data (Fig 1B), and this isoform encodes four predicted transmembrane domains (Fig 1C). RT-PCR of wild-type L4 stage worms further confirms that the gene is expressed at detectable levels, and that exon three is alternatively spliced (Fig 1D). We have submitted this information to WormBase, and the new gene is set to appear as WBGene00306123 (transcripts *Y97E10C.2a* and *Y97E10C.2b*) in release WS283.

While inspecting this locus, we also noted RNA-seq evidence for the gene immediately upstream (*T05B11.7*) that deviates from its annotated gene model. The RNA-seq data indicate that prior to reaching the annotated stop codon in exon three, the mRNA is spliced to an unannotated downstream exon (Fig 1E). Therefore, the annotated stop codon is in fact located within an intron, and the *T05B11.7* gene contains four exons. This increases the coding sequence of *T05B11.7* from 852 to 924 nucleotides. We also found evidence for alternative splicing in the *T05B11.7* gene. Exon one harbors an alternative 5’ splice site which is used in a small fraction of the transcripts detected by RNA-seq (Fig 1F) and which is also detectable via RT-PCR (Fig 1G). These alternative isoforms (including the new addition of exon four) will appear in WS283 as transcripts *T05B11.7*a and *T05B11.7*b.

Finally, the gene *T05B11.7* is currently annotated as part of an operon (CEOP5584) along with its upstream gene *clic-1* (Fig 1H). Our newly-described gene *Y97E10C.2*, which is located immediately downstream of *T05B11.7*, is now included as the final gene in this operon, making CEOP5584 a 3-gene operon (Fig 1H).

## Methods

**Analysis of RNA-seq data:** Wild-type RNA-seq data used in this manuscript were previously published (Choudhary *et al.*, 2021; Norris *et al.*, 2017, 2014) using polyA-selected RNA obtained from either L3-stage or L4-stage wild-type (N2) hermaphrodites. In our initial search for alternative splicing which led to the identification of gene *Y97E10C.2*, we performed differential splicing analysis using JUM (Wang and Rio, 2018) on STAR-mapped (Dobin *et al.*, 2013) short read (150 bp paired end) sequencing, stipulating that to be considered an alternative splicing event, both alternative junctions must be represented by at least 5 unique junction-spanning reads in at least two out of the three biological wild-type replicates tested.

**RT-PCR:** RT-PCR was performed on total RNA extracted from L4-stage wild-type (N2) hermaphrodites. Primers for *Y97E10C.2:* CGAGTCAAACTGAGCATGTG & AACACTCCACCAACAAGTAGAC*.* Primers for *T05B11.7:* TGTGCAACGGCAGCAAGAAG & CCAACAGTTTCCAGCTCCGAATC.
